# Knowledge and Use of Zinc Supplementation in the Management of Childhood Diarrhoea among Health Care Workers in Public Primary Health Facilities in Benin-City, Nigeria

**DOI:** 10.5539/gjhs.v4n2p68

**Published:** 2012-03-01

**Authors:** Vivian O. Omuemu, Ifeanyi J. Ofuani, Itse C. Kubeyinje

**Affiliations:** Department of Community Health, College of Medical Sciences University of Benin, PMB 1154, Benin-City, Edo State, Nigeria

**Keywords:** Zinc supplementation, Childhood diarrhoea, Health care providers

## Abstract

Zinc supplementation reduces the severity, duration and recurrence of childhood acute diarrhoea. These beneficial effects of zinc in the treatment of diarrhoea led to the inclusion of a 10-14 days treatment regimen by the WHO/UNICEF. This study assessed the level of knowledge and use of zinc supplementation in the management of childhood diarrhoea among health care workers in public primary health facilities in Benin-City, Nigeria.

**Methodology:**

This cross-sectional study was carried out among the total population of health care providers in public primary health facilities in Benin-City. Data collection was done using a pre-tested, structured, self-administered questionnaire and data was analyzed using SPSS version 15.0.

**Results:**

A total of 168 health care workers participated in the study. Two-thirds of them were aware of zinc supplementation but specific knowledge of zinc supplementation in the management of childhood acute diarrhoea was poor. Thirty-five percent of them prescribed zinc when managing childhood diarrhoea and only 10% of these do so for every case of childhood diarrhoea. About 84.6% of them prescribed the correct dose of zinc while less than half of them prescribe it for the correct duration. All but one of them prescribed zinc in addition to ORS in line with the WHO guideline.

**Discussion:**

The study revealed a gap in the knowledge and practice of use of zinc supplementation in the management of childhood diarrhoea. It is recommended that nationwide campaigns should be embarked on to promote the use of zinc supplementation in the clinical management of childhood diarrhoea.

## 1. Introduction

Diarrhoea which is the passage of three or more loose stools per day continues to be one of the leading causes of morbidity and mortality in under-five children in low- and middle-income countries including Nigeria (Black *et al.*, 2010). Death from acute diarrhoea is often caused by dehydration and malnutrition mainly due to loss of nutrients from the body during episodes of diarrhoea. Though the introduction of Oral Rehydration Salt (ORS), implementation of routine Vitamin A supplementation and measles vaccine, improved breast feeding and continued feeding, improved sanitation, access to clean water and hand-washing led to a decline in diarrhoea mortality from an estimated 4.5 million deaths in the early 1980s to 1.3 million in 2008, acute diarrhoea continues to be a burden on children particularly in developing countries (Black, *et al.*, 2010; WHO/UNICEF, 2009).

Following the evidence of the clinical benefits of zinc supplementation as an adjunct therapy to ORS in the management of diarrhoea, the World Health Organization (WHO) and United Nations Children’s Fund (UNICEF) included zinc supplementation in the treatment of acute diarrhoea in May 2004. This guideline includes the use of low osmolarity ORS and a 10-14 days treatment with 10mg per day of zinc tablets for infants under 6 months and 20mg per day of zinc tablets for older children (WHO/UNICEF, 2004).

Zinc is an important micronutrient which plays a critical role in cellular growth and function of the immune system (Indian Academy of Paediatrics, 2007; WHO, 1996). Zinc becomes depleted during diarrhoeal episodes and these intestinal losses aggravate any pre-existing zinc deficiency (Indian Academy of Paediatrics, 2007). Numerous clinical trials have shown that zinc tablets given as an adjunct to Oral Rehydration Therapy (ORT) in the treatment of diarrhoea reduces the duration and severity of the diarrhoeal episodes as well as decreases the occurrence of diarrhoea in the 2-3 months following the episode (Bhutta *et al.*, 2000; Bhatnagar *et al.*, 2004; Baqui *et al.*, 2002; Strand *et al.*, 2002). Addition of zinc to current case management of acute diarrhoea has also been reported to lead to reduction in the use of antibiotics and increase in the rate of use of Oral Rehydration Salt (ORS) (Baqui *et al.*, 2004; Bhandari *et al.*, 2005).

Success in reducing morbidity and mortality due to diarrhoea depends on the acceptance of these benefits by government, policy makers and the medical community. In Nigeria, findings from localized clinical and community studies in some parts of Nigeria revealed that zinc supplementation caused a reduction in the severity, duration and recurrence of childhood acute diarrhoea (Nigeria Zinc Study Team, 2008). Following this, an operational research was undertaken to assess the feasibility of distribution of zinc tablets by health care workers for the management of diarrhoea in under-five children and to have an update of the knowledge and skills of selected health care workers – State Nutrition Officer, Control of Diarrhoeal Disease/Acute Respiratory Tract Infection (CDD/ARI) Officer, Local Government Area Nutrition focal person and Officers in charge of the Primary Health Care facilities and their assistants. These health care workers received cascaded training from the zonal level to the local level (Nigeria Zinc Study Team, 2008).

The awareness of the inclusion of zinc in the management of childhood diarrhoea among health care providers has been reported to be high in some developing countries (Larson *et al.*, 2009). Despite this growing awareness, its use in diarrhoea treatment in many of these countries has lagged behind (Fisher-Walker *et al.*, 2009; Hoekstra, 2001). Measures aimed at improving the level of knowledge and use of zinc supplementation among health care providers in the management of childhood diarrhoea would be expected to reduce the morbidity and mortality from childhood diarrhoea. This would also improve the overall child health indicators and contribute to the achievement of the fourth Millennium Development Goal.

This study was designed to determine the level of knowledge and use of zinc supplementation in the management of childhood diarrhoea among health care workers in public primary health facilities in Benin-City, Nigeria.

## 2. Materials and Methods

### 2.1 Research Design and Setting

This study carried out among health care workers in Benin-City between January and June 2010 was cross-sectional and descriptive in design. Benin-City is the capital of Edo State in the Southern part of Nigeria with a population of 1,086,882. The city is metropolitan and comprises three Local Government Areas (Oredo, Egor and Ikpoba-Okha). There are 31 Primary Health Care (PHC) Centres in Benin-City.

### 2.2 Participants and Data Collection

The total population of 182 health care providers in the PHC facilities in Benin-City was eligible to participate in the study. Data was collected with the aid of a pre-tested, structured, self-administered questionnaire which comprised of open and close-ended questions. Information was sought on socio-demographic characteristics, knowledge, attitude and practice with respect to zinc supplementation in the management of childhood acute diarrhoea. Correct knowledge and practice of dosage and duration of administration of zinc in diarrhoeal management was as recommended by the WHO (WHO/UNICEF, 2004). Institutional approval to carry out the study was sought and obtained from the Local Government Authority and verbal informed consent was sought and obtained from the participants.

### 2.3 Data Analysis

Data analysis was by computer using the SPSS version 15.0. Simple proportion was calculated to determine the level of knowledge and use of zinc. Data were presented on tables and association between categorical variables assessed using Χ^2^ test and level of significance set at p-value less than or equal to 0.05.

## 3. Results

A total of 168 out of 182 health care workers completed and returned their questionnaire giving a response rate of 92.3 percent. Their mean age was 36.3 ± 9.1 years (95 CI = 34.9-37.7). Their demographic characteristics are shown on table 1.

**Table 1:** Demographic characteristics of respondents

Characteristics	Frequency (N = 168)	Percent
**Age group (years)**		
20-29	50	29.8
30-39	55	32.7
40-49	54	32.1
50-59	9	5.4
**Sex**		
Male	27	16.1
Female	141	83.9
**Categories of HCWs**		
CHEWs	95	56.5
Nurses/Midwives	59	35.1
CHO	9	5.4
Doctors	5	3.0
**Duration of practice (years)**		
1-5	51	30.3
6-10	44	26.2
11-15	22	13.1
16-20	27	16.1
>20	24	14.3

HCWs- Health Care Workers

CHEWs- Community Health Extension Workers

CHOs- Community Health Officers

One hundred and eleven (66.1%) of the health care workers were aware of zinc supplementation and their major sources of information were from colleagues (50.5%) and training workshop (39.6%). About 70.4% of the male health care workers were aware of zinc compared with 65.2% of the female health care workers but the difference was not statistically significant, Χ^2^ = 0.265, df = 1, p = 0.607. The doctors (100%) had a significantly higher proportion of those who were aware of zinc supplementation compared with the other categories of health care workers, Χ^2^ = 15.844, df = 2, p = 0.001.

Specific knowledge of zinc supplementation with respect to management of acute diarrhoea by health care workers is shown on table 2. About 50.5% and 32.4% of them knew that persistent and acute diarrhoea in children, respectively can be treated with zinc. Benefits of zinc supplementation in diarrhoea management mentioned by them included reducing severity of diarrhoea (57.7%), reducing duration of diarrhoea (34.2%) and preventing future episodes of diarrhoea (16.2%). The highest proportion of them (66.7%) knew the correct dosage and duration of administering zinc supplements during a diarrhoea episode. Majority (85.6%) of the health care workers knew that zinc supplementation was an adjunct to Oral Rehydration Salt (ORS) in diarrhoea management.

**Table 2:** Specific knowledge about zinc supplements

Variable	Frequency (N = 111)	Percent
**Knowledge of types of diarrhoea treated with zinc supplements:**		
Persistent diarrhea	56	50.5
Acute watery diarrhoea	36	32.4
Dysentery	6	5.4
Cholera	13	11.7
Don’t know	10	9.0
**Knowledge of benefits of zinc supplements:**		
Reduces severity of diarrhea	64	57.7
Reduces duration of diarrhea	38	34.2
Prevents future episodes of diarrhea	18	16.2
Keeps children strong	2	1.8
Keeps children healthy.	1	0.9
Don’t know	11	9.9
**Knowledge of dosage and duration of administering zinc supplements:**		
Correct knowledge	74	66.7
Incorrect knowledge	20	18.0
No knowledge	17	15.3
**Knowledge of zinc as an adjunct to ORS in diarrhea management:**		
Correct knowledge	95	85.6
Incorrect knowledge	1	0.9
No knowledge	15	13.5

Majority of the respondents 96 (86.5%) mentioned that zinc supplementation was safe for use in children while 5 (4.5%) said it was not safe. Ten (9.0%) of them had no idea about the safety of zinc supplementation in children. All those who reported that zinc was not safe for use in children thought so because it causes vomiting.

Majority of the health care workers (97.3%) had no reservations towards prescribing zinc to children while 74.8% thought zinc supplementation should replace previous forms of diarrhea treatment. Forty-four (39.6%) of the health care workers had received training on zinc supplementation in management of childhood diarrhoea while the rest had not.

Thirty-nine (35.1%) of the health care workers prescribe zinc when managing childhood diarrhoea. A higher proportion of the female health care workers (38.1%) prescribed zinc more than the male health care workers (21.1%) but this was not statistically significant, Χ^2^ = 1.995, df = 1, p = 0.159. The Community Health Officers-CHOs (62.5%) prescribed zinc more than the other categories of health workers, Χ^2^ = 22.923, df = 2, p = 0.001. Those who had received training on zinc supplementation (56.8%) prescribed zinc more than those who had no training (20.9%), Χ^2^ = 15.038, df = 1, p = 0.001.

**Table 3:** Prescription of zinc supplements by respondents in diarrhoea management

CHARACTERISTICS	PRES YES	CRIP NO	TION TOTAL
**Sex**			
Male	4 (21.1)	15 (78.9)	19 (100.0)
Female	35 (38.0)	57 (62.0)	92 (100.0)
	Χ^2^ = 1.995	df = 1	p = 0.159
**Category of HCWs**			
CHEWs	6 (11.8)	45 (88.2)	51 (100.0)
Nurses/Midwives	25 (53.2)	22 (46.8)	47 (100.0)
CHO	5 (62.5)	3 (37.5)	8 (100.0)
Doctors	3 (60.0)	2 (40.0)	5 (100.0)
	Χ^2^ = 22.923	df = 2	p = 0.001*
**Received formal training on zinc.**			
Yes	25 (56.8	19 (43.2)	44 (100.0)
No	14 (20.9)	53 (79.1)	67 (100.0)
	Χ^2^ = 15.038	df = 1	p = 0.001*
**TOTAL**	39 (35.1)	72 (64.9)	111 (100.0)

* Statistically significant

Of the 39 health care workers who prescribed zinc, 4 (10.3%) always prescribed it for every case of childhood diarrhea while the rest 35 (89.7%) did so occasionally. Nineteen (48.7%) of those who prescribe zinc did so for the correct duration and 33 (84.6%) prescribe the correct dose. Majority 38 (97.4%) of those who prescribed zinc prescribe it in addition to ORS while 1 (2.6%) health worker prescribed antibiotics in addition to ORS and zinc for diarrhea management. Thirty seven (94.9%) of those who prescribed zinc counseled mothers/caregivers on the proper way to administer zinc and 17 (43.6%) paid follow-up visits to monitor the children. Thirteen (41.9%) of the 31 PHC facilities in Benin-City had zinc tablets in stock at the time of the survey.

## 4. Discussion

The study showed that a high proportion of the health care workers were aware of zinc supplementation for managing childhood diarrhoea and the higher proportion of them heard from their colleagues than from a training workshop. This is because the training workshop organized by the government in 2007 involved some selected health workers from each facility and was in turn expected to train other health care workers in their facilities.

Knowledge of specific type of diarrhoea where zinc supplementation is indicated was poor. Adequate knowledge would ensure appropriate prescription of zinc in diarrhoeal cases and prevent irrational prescription. Furthermore, majority of the health care workers also knew at least one benefit associated with the use of zinc supplementation when managing diarrhoea. This corroborates what has been reported elsewhere (Nepal Family Health Programme, 2006) and adequate knowledge of its benefits would influence the health care workers to prescribe it to children who present with diarrhoea.

It is also worth noting that about two-thirds of the health care workers who were aware of zinc knew the correct dosage and duration of administering zinc during a diarrhoeal episode in line with the WHO/UNICEF guidelines (WHO/UNICEF, 2004). This is in contrast to what was reported in Nepal where all the health care providers knew the correct dosage and duration of zinc. This higher proportion could be attributed to the fact that all the health workers had received formal training (Nepal Family Health Programme, 2006).

The fact that majority of the health care workers knew that zinc supplementation was safe when administered to children would encourage them to prescribe zinc for cases of childhood diarrhoea. Evidence has shown that zinc treatment is effective and generally safe (Roy, *et al.*, 1997). The only known side effect of zinc supplementation is vomiting which is rarely reported and is typically attributed to a metallic taste in the tablet. It has been reported also that the use of high quality tablets can avert this side effect (Roy, *et al.*, 1997; Khan, *et al.*, 2007).

Generally, the health care workers had a positive attitude towards zinc supplementation as an adjunct to ORS in management of acute diarrhoea. Positive attitude would encourage them to endorse and promote zinc supplementation for case management of acute diarrhoea in children.

The small proportion of health care workers in this study who had received formal training on the use of zinc supplementation in the management of childhood diarrhoea is cause for concern. Training workshops for health care providers present an excellent opportunity to review the basic diarrhoea control strategies in addition to the current guidelines by the WHO (The USAID Micronutrient Programme, 2011). This is particularly most important for PHC workers who are usually the first level of contact with individuals and the community, and have a unique one-on-one opportunity to influence compliance to treatment recommendations by informing and teaching mothers/caregivers home-based diarrhoea therapy including zinc supplementation and ORS use.

The study also showed that only one-third of the health care workers prescribe zinc when managing diarrhoea in children and this is lower than reported in Benin Republic where nearly all (96.0%) of the health care providers prescribed zinc (Macdonald, *et al.*, 2010).

Expectedly, those who had received formal training on the use of zinc supplementation in management of childhood diarrhoea prescribed it more. This has been reported elsewhere and highlights the importance of formal training on the use of zinc therapy (Macdonald, *et al.*, 2010).

Though majority of the health care workers prescribed the correct dose of zinc, less than half of them did so for the correct duration. This finding highlights the importance of ensuring that communication messages focus on the basic but key information about correct use of any product (Wang, *et al.*, 2011). Prescription of zinc to children with diarrhoea at the appropriate dose and duration would enhance and ensure maximal effect of zinc on cases of diarrhoea (Bhutta, *et al.*, 2000; Bhatnagar, *et al.*, 2004; Baqui, *et al.*, 2002; Strand, *et al.*, 2002). Consequently, this reduces the need for hospitalization and additional treatment, both of which can be expensive for families.

It is encouraging to note that all but one of the health care workers prescribed zinc in line with the WHO/UNICEF guideline for management of acute diarrhoea. This is in contrast to findings from Benin Republic where majority of the health care providers (86.0%) prescribe an antibiotic in addition to zinc (Macdonald, *et al.*, 2010). Evidence has shown that 88 percent of diarrhoeal deaths can be prevented with widespread use of low osmolarity ORS and zinc (Jones, *et al.*, 2003). The use of antibiotic in managing cases of acute diarrhoea in children was reported by only one respondent which further buttresses the need for education of health care workers to ensure adherence to treatment guidelines. Education has been proven to lead to an increase in the use of zinc and decline in the use of antibiotics for case management of acute diarrhoea (Balasubramanian & Ganesh, 2008; Bhandari, *et al.*, 2008).

Majority of the health care workers who prescribed zinc counseled the caregivers on the appropriate use of zinc but less than half of them paid follow-up visits to monitor the children. This is in agreement with reports from Mali (Gilroy, *et al.*, 2005). Adequate counseling and follow-up of patients would ensure that correct dosage regimen for diarrhoeal treatment is adhered to by caregivers (Wang, *et al.*, 2011).

Furthermore, less than half of the Primary Health Care centres had zinc tablets in stock at the time of the study. This may reflect poor financial and logistics support from the government. Since the prevalence of childhood diarrhea is still high, the demand for zinc supplementation is expected to be high. It is therefore, necessary to ensure adequate supply and availability of zinc tablets to all health facilities and health care providers including those in the grassroots. This will require the commitment of government at all levels to provide financial backing for procurement and distribution of zinc tablets.

## 5. Conclusion

The study has revealed a gap in the knowledge and practice with respect to zinc supplementation in the management of childhood diarrhoea. It is recommended that nationwide social marketing campaigns involving all stakeholders be embarked on to promote the use of zinc supplementation in the clinical management of childhood diarrhoea.

## Limitation of Study

The potential for recall bias exists in this study since data were based on self-reported information. In addition, being a cross-sectional study, a causal relationship between specific variables and use of zinc supplementation cannot be inferred.
